# Changing the system of psychiatric care in bulgaria. Recover-E project in bulgaria

**DOI:** 10.1192/j.eurpsy.2021.201

**Published:** 2021-08-13

**Authors:** V. Nakov

**Affiliations:** Department Mental Health, National Center of Public Health and Analyses, Sofia, Bulgaria

## Abstract

**Introduction:**

Bulgaria has undergone a number of very significant political and social changes over the past 150 years that has also impacted on the delivery of mental health care. There has been a 40% reduction in the number of inpatient psychiatric beds in Bulgaria within the past 20 years leading to the current state of approximately European average bed numbers per 100 000 population. This does not appear to have been accompanied by an increased investment in ambulatory / outpatient mental health services.

**Objectives:**

Description of the advantages and disadvantages of mental health services in Bulgaria, available staff and distribution in the country. The project RECOVER-E and its activities in Bulgaria are described.

**Methods:**

Sources of health statistics of Bulgaria are used and analyzed. Maps and tables were used for visualization.

**Results:**

Taking into account the situation described in this way and the EPA guidelines for change in the system, a mental health strategy has been proposed.

**Conclusions:**

It has a long and significant legacy of underfunding of mental health services, which has undoubtedly caused significant economic damage to Bulgaria through surmountable results increasing health and social care costs, and surmountable loss of economic productivity. A significant increase in the budget allocated to mental health and related social care services.

**Disclosure:**

No significant relationships.

